# Quantitative Cardiac Positron Emission Tomography: The Time Is Coming!

**DOI:** 10.6064/2012/948653

**Published:** 2012-08-27

**Authors:** Roberto Sciagrà

**Affiliations:** Department of Clinical Physiopathology, Nuclear Medicine Unit, University of Florence, Largo Brambilla 3, 50134 Florence, Italy

## Abstract

In the last 20 years, the use of positron emission tomography (PET) has grown dramatically because of its oncological applications, and PET facilities are now easily accessible. At the same time, various groups have explored the specific advantages of PET in heart disease and demonstrated the major diagnostic and prognostic role of quantitation in cardiac PET. Nowadays, different approaches for the measurement of myocardial blood flow (MBF) have been developed and implemented in user-friendly programs. There is large evidence that MBF at rest and under stress together with the calculation of coronary flow reserve are able to improve the detection and prognostication of coronary artery disease. Moreover, quantitative PET makes possible to assess the presence of microvascular dysfunction, which is involved in various cardiac diseases, including the early stages of coronary atherosclerosis, hypertrophic and dilated cardiomyopathy, and hypertensive heart disease. Therefore, it is probably time to consider the routine use of quantitative cardiac PET and to work for defining its place in the clinical scenario of modern cardiology.

## 1. Introduction

At the beginning of its clinical use in the late eighties of the last century, positron emission tomography (PET) was mainly proposed for neurological and cardiological indications. At that time, ^18^F-labelled deoxyglucose (FDG) was regarded as an effective tracer for studying brain glucose consumption and for identifying viable hibernating myocardial tissue in patients with congestive heart failure of ischemic etiology [1]. Effective myocardial perfusion tracers such as ^13^N-labelled ammonia, ^15^O-labelled water, and Rubidium-82 (^82^Rb), a potassium-analogue, were also available [2–6]. The intrinsic advantages of PET over single-photon emission computed tomography (SPECT) were particularly attractive for heart imaging, because the possibility to perform an effective attenuation correction permitted to overcome a main limitation of SPECT perfusion imaging [3, 4, 6]. Similarly, the metabolic imaging with ^18^FDG had no counterpart within SPECT tracers [7]. However, in spite of several comparative studies that demonstrated the superior diagnostic performance of PET over SPECT for perfusion imaging and the wide literature about viability using flow-metabolism imaging with any perfusion tracer plus ^18^FDG, the clinical use of PET in cardiology did not achieve the expected dissemination. Several practical considerations hampered its use: first of all, naturally, the need of on-site cyclotron for tracer production. Even the availability of a generator-produced perfusion tracer like ^82^Rb could not overcome the problem, because the exceedingly high costs did not justify its purchase in the absence of a very high patient throughput. Finally, in many countries the reimbursement issue remained unsolved. 

In the following years, the appearance of gated SPECT reinforced the role of traditional nuclear cardiology and improved its diagnostic and prognostic capabilities [8, 9]. Simultaneously, the exponential growth of oncological indications limited the availability of PET scanners for other purposes. Therefore, PET did not reach a widespread clinical use for heart disease. On the other hand, the intrinsic quantitative capabilities of PET were applied in a large number of research studies that explored their value in a broad spectrum of clinical conditions. 

Presently, the wide diffusion of PET facilities (often with on-site cyclotron) supported by oncology, the enhanced availability of ^82^Rb generators, and the imminent marketing of ^18^F-labelled perfusion tracers have renewed the interest for the issue of a generalized use of PET in cardiology. As a proof of this, the relative merits of PET and SPECT and the problems related to the incorporation of PET in the clinical practice have been most recently reviewed [10–12]. However, the most important reason to support the widespread use of cardiac PET is still the same that had been identified 20 years ago by M.M. Ter-Pogossian:

*“The (…) utility of PET in cardiovascular research and in clinical cardiology results from its quantitative power and applicability to assessment of the kinetics in tissue of physiologic labeled substrates and tracers. (…) Because of its quantitative power (…) PET provides information delineating biochemical and physiological processes that is not available by any other means.” [13].*

Therefore, it would be desirable to get this opportunity to orient the renewed interest for cardiac PET in the direction of a quantitative approach to data interpretation, in order to exploit the full and most specific potential of this methodology. Aim of this paper is to describe the added value and the clinical role of quantitation in cardiac PET to support the expansion of this imaging modality and the routine application of PET measurements in the clinical practice. 

## 2. Technical Considerations in Quantitative Cardiac PET

To be used for quantitation, PET must be of optimal quality. Therefore, all the technical requirements of high-quality PET imaging must be fulfilled. Although PET has the advantage of an inherently effective attenuation correction, which is nowadays usually performed with the computed tomography (CT) images acquired using a PET/CT scanner [14], several factors may degrade PET images and accordingly affect the accuracy of quantitative measurements. The most important are related to the intrinsic nature of positron emission, such as the positron range, that is, the distance that a positron travels before undergoing annihilation, and the circumstance that the two emitted photons are not perfectly collinear; then there are scatter and random coincidences; various algorithms have been implemented in the reconstruction programs to correct whenever possible these interferences [15–20]. Attenuation correction itself can cause artifacts if not properly performed, for instance because of patient motion, and there are various methods to deal with the possible misalignment problems [21–23]. Other technical improvements of PET that favorably influence its quantitative capabilities are 3D acquisition, the availability of better detector materials, the time-of-flight technology, the option to acquire dynamic sequences in list mode instead of using frames of predefined duration, the implementation of electrocardiographic, and possibly of respiratory gating [24–27]. The image reconstruction has been improved by the use of iterative algorithms; although their advantage over the standard filtered back projection for the issue of quantitation is not yet definitively established [28, 29]. 

## 3. Tracers for Quantitative Cardiac PET

Both perfusion and metabolic tracers are available for quantitative cardiac PET. For the measurement of myocardial blood flow (MBF) at rest and under stress and for the calculation of the coronary flow reserve (CFR), which is the ratio of stress over resting MBF, three tracers have been extensively used and evaluated: ^15^O-water, ^13^N-ammonia, and ^82^Rb. More limited is the experience with ^11^C-acetate: because of its peculiar kinetics, this tracer allows the simultaneous measurement of MBF and of myocardial oxygen consumption, and apparently is superior to ^13^N-ammonia in image quality and statistical accuracy of quantitative data [30–33]. However, ^11^C requires an on-site cyclotron, and particular care in target handling and tracer synthesis because of its gaseous nature, so that its use in the clinical practice has never got wide acceptance [34]. An additional perfusion tracer labeled with ^18^F is under advanced clinical evaluation and will be likely available in the near future [35]. For quantitative imaging of glucose metabolism there is ^18^FDG [36, 37]. Metabolic and neuronal tracers labeled with ^11^C have been used as well, but their availability is limited and their role in clinical imaging is still undefined [37, 38]. 

The features of the commonly employed perfusion tracers are summarized in Table 1. None of them can be regarded as definitely perfect. There are no doubts that in terms of extraction a diffusible tracer such as ^15^O-water is ideal [39–44]. However, the very short half-life and the immediate washout from myocardial tissue preclude the achievement of qualitatively acceptable images for visual assessment, so that the study evaluation is exclusively based on the MBF measurements [25, 43, 44]. Furthermore, not all on-site cyclotrons are able to produce ^15^O-water. Conversely, the production and handling of ^13^N-ammonia are much simpler and this can be regarded as the most reasonable tracer for all centers with an on-site cyclotron [25, 34]. The combination of visually evaluable images for an easy region-of-interest definition, quite high myocardial extraction, defined tracer kinetics, and wide clinical experience make ^13^N-ammonia probably the best routine tracer for quantitative cardiac PET to this point available [25, 43–52]. The simple use of myocardial distribution images for the qualitative comparison of rest and stress tracer uptake, although employed with good results in the clinical practice, does not make the most of the full potential of this tracer [53]. As regards ^82^Rb, its main advantages are that it is a generator-produced tracer and there is a large clinical experience on its use [43, 44]. On the other hand, the image quality is lower than that of ^13^N-ammonia both because of the shorter half-life and of the high energy of the positrons, with consequent long path from site of emission to site of annihilation [43]. Furthermore, the extraction fraction is suboptimal and the kinetics model less established [43, 44]. Nevertheless, many recent papers clearly indicate that ^82^Rb quantitative PET is a well feasible and reliable technique [54–58]. The present availability of ^82^Rb generators produced by vendors with worldwide distribution is an additional prerequisite for the more extensive use of this methodology [25, 59, 60]. 

## 4. Quantitative Measurements in Cardiac PET

The largest experience in cardiac quantitative PET is in the measurement of MBF. There are a large number of different approaches, mainly based on compartmental models of tracer kinetics [61]. In general, they require a dynamic acquisition starting at the moment of tracer injection, which is then prolonged until the phase of tracer uptake in the myocardium is completed. By the identification of volumes of interest drawn on the ventricular cavities and on the myocardial wall of the left ventricle, it is possible to obtain time activity curves depicting the input of the tracer and its accumulation within the myocardial tissue. These time activity curves are then properly fitted and the related parameters calculated, obtaining the tracer input function and the tracer amount within the myocardium. According to the chosen compartmental model, these values are included in the equations for the calculation of the kinetic parameter that best depicts MBF. The equations often include appropriate correction factors for other variables, such as activity spill over from blood into the myocardium, as well as for the partial volume effect and the system resolution [62–64]. The technical details of these procedures go beyond the aim of this paper, and can be found in several methodological papers and outstanding reviews [43, 44, 59]. Just as an example, the algorithm proposed by Lortie et al. for MBF calculation using ^82^Rb will be shortly described [58]. They use for describing ^82^Rb kinetics a one-compartment model:

(1) $$\begin{array}{c} C_{m}\left(t\right)=K_{1}e^{-k_{2}t}*C_{a}\left(t\right), \end{array}$$

where *C*_*a*_(*t*) and *C*_*m*_(*t*) are the concentrations of ^82^Rb in arterial blood and myocardium, respectively. To address spill over and partial volume effect, a correction is introduced:

(2) $$\begin{array}{c} C_{\text{PET}}\left(t\right)=F_{LV}C_{LV}\left(t\right)+\left(1-F_{LV}\right)C_{m}\left(t\right), \end{array}$$

where *C*_PET_ is the measured concentration of ^82^Rb, *F*_*LV*_ a number between 0 and 1 and (1 − *F*_*LV*_) an estimate of partial volume recovery coefficient [64]. After that the parameters are estimated using a weighted least-squares method, *K*_1_ values are converted to MBF taking into account the flow-dependent ^82^Rb extraction:

(3) $$\begin{array}{c} K_{1}=\left(1-a\times e^{-b/\text{MBF}}\right)\times \text{MBF}, \end{array}$$

where *a* and *b* are correction factors derived from comparison with ^13^N-ammonia MBF using a weighted least-squares algorithm. 

Many studies have compared the reliability of the different models used for the quantitation of MBF and have shown that there are methodological inequalities that must be taken into account if the results of diverse laboratories have to be compared [49, 61, 65]. What is most important for the clinician, however, is that nowadays there is a large choice of programs for performing in a user-friendly way this kind of procedures [66–70]. Although they have not reached the diffusion and the implementation degree of the gated SPECT analysis programs, they are nevertheless easily accessible. Some of them have been already implemented in PET scanners by vendors, others can be purchased from software producers, and finally some have been developed in academic institutions and either commercialized or made freely available on the net. Yet, the results they achieve, also because of the different models that can be used, are not always exchangeable. However, various studies demonstrate that the reproducibility of MBF calculated using the same method is excellent [66–75]. 

As regards glucose consumption, the most practiced approach is the graphical analysis as proposed by Patlak [76]. Traditionally, ^18^FDG imaging has been performed with a visual (or semiquantitative) evaluation of tracer uptake after glucose loading for the detection of increased uptake in regions with perfusion defects (flow/metabolism mismatch) and this method has been considered a noninvasive gold standard for the identification of viable myocardium within dysfunctional ventricular segments [1, 7]. More recently, however, several studies have shown that the measurement of the glucose metabolic rate under more physiologic fasting conditions has an important meaning in different clinical settings [77, 78]. Therefore, it can be expected that quantification of ^18^FDG will be increasingly used.

## 5. MBF and CFR in Various Clinical Settings

Aim of the following section is to report the many studies that have investigated MBF and CFR in health and disease. Most of these studies have been performed on small patient populations. However, their results are consistent in showing that quantitative measurements are highly effective for detecting significant differences related to the explored condition. First of all, the behavior of MBF and CFR in normal subjects has been examined. Although minor differences exist, mainly related to the tracer and the calculation model, the values appear quite homogeneous, with baseline mean resting values ranging from 0.8 to 1.2 mL/min/g tissue and mean stress values (under coronary vasodilator, adenosine, or dipyridamole) ranging from 2.7 to 4.6 mL/min/g tissue [41, 47, 79–85]. Accordingly, the mean CFR ranges from 2.9 to 4.4 mL/min/g tissue [41, 47, 79–85]. The baseline values are influenced by the oxygen demand and thus the correction of the resting MBF for the rate pressure product has been proposed in order to obtain a reliable CFR [47, 71, 85–87]. Age influences MBF through the changes in cardiac work [85]. On the other hand, a reduction in hyperemic MBF has been registered with ageing [88]. These changes cause a decrease in CFR in older subjects [89]. As regards gender, some reports indicate higher MBF values at rest and during hyperemia in women, with a possible relationship with the better blood lipid profile [87, 90]. In another most recent report, it has been observed that among normal subjects those with potential risk factors such as familial history of coronary artery disease (CAD) and slight abnormalities in blood lipids have lower MBF values than “true” normals, that is, subjects with completely negative familial history and perfectly regular blood profile [91].

A series of studies has explored MBF in relationship with various conditions that could affect it. In syndrome X, considered as a paradigm of potential ischemia in the absence of obstruction of the epicardial vessels, the various studies have not demonstrated a clear-cut difference in stress MBF values between patients and healthy controls: although a quite large proportion of syndrome X patients have a reduced CFR, there is an overlap with the normal subjects and no relationship could be demonstrated between CFR abnormalities and symptoms or ischemia-like electrocardiographic changes [80, 92–95]. In the broader category of patients with typical angina and normal coronary angiograms, the large majority has an abnormal CFR, that is, caused both by increased resting MBF and decreased stress MBF [80, 96]. More recently, a relationship between the presence of CFR abnormality and worse risk factor profile has been reported in these subjects [97]. 

Other studies have examined the behavior of MBF and CFR in patients with various risk factors for CAD. In hypertensive patients, another group in whom ischemic symptoms are frequently reported without detectable coronary angiographic abnormalities, MBF has been found decreased even in subjects without signs of left ventricular hypertrophy, and improvements have been demonstrated after antihypertensive therapy [98, 99]. In particular, the improvements may precede the antihypertensive effect and are partly related to the drug category [99, 100]. On the other hand, in patients with left ventricular hypertrophy, lower MBF and CFR have been found as compared with age-matched controls, and the difference is larger in those with primary hypertrophy (hypertrophic cardiomyopathy-HCM) than in those with secondary hypertrophy (caused by hypertension or by aortic stenosis); in this last group there is still a quite good hyperemic response, but this is partly offset by the high resting MBF [101, 102]. Another very interesting finding is that even in young patients fulfilling the definition of borderline hypertension there is a CFR reduction as compared to age-matched controls: this would imply that an impaired vasodilator capacity can be a most precocious sign of hypertension-related myocardial damage [103].

In diabetic patients, it has been demonstrated that there is a reduction in CFR in young subjects without other evidence of cardiac involvement and with no or just minimal signs of vasculopathy in other organs [104]. In noninsulin dependent diabetics, male sex, and poor glycemic control, but not age and type of therapy, have been found related to the degree of microvascular impairment [105]. By adding to the vasodilator stress the cold pressor test (CPT), which explores the endothelial reactivity to sympathetic stimulation, Di Carli et al. observed that together with a reduced vasodilator reserve, diabetic patients with neuropathy have an abnormal response to CPT, in agreement with other signs of sympathetic nerve dysfunction [106]. In a larger patient population, the abnormal response to CPT has been registered in approximately one third of asymptomatic diabetics and associated with higher baseline MBF [107]. In premenopausal women, those with diabetes have a reduced CFR and an abnormal CPT response compared with healthy controls, and their values are similar to those observed in postmenopausal women, showing one mechanism through which diabetes abolishes the sex differential in CAD [108]. Apparently, both endothelium-dependent and -independent coronary vasodilator function abnormalities are related to the chronic hyperglycemia and not to the type of diabetes (insulin-deficient versus insulin-resistant) [109]. However, other authors have registered an impaired CPT response in insulin-resistant patients [110]. Moreover, an increasing severity of microvascular dysfunction has been observed in patients with type 2 diabetes, starting from those with insulin resistance but without glucose intolerance, to those with isolated impaired glucose tolerance, normotensive diabetes, and hypertension plus diabetes [111]. 

The capability of different treatments to correct MBF abnormalities in diabetic has been tested. Bengel et al. have demonstrated that the improved glycemic control using an insulinotropic agent does not influence the MBF values at rest, under adenosine and during CPT [112]. Conversely, in diabetic patients with CAD, insulin infusion improves resting and adenosine MBF both in ischemic and nonischemic regions [113]. In the long term, the achievement of euglycemia is associated with a significant improvement of the MBF response to CPT [114]. The same group has been able to demonstrate a slower progression of the coronary vessel structural alterations present at baseline in diabetics, after an effective glucose-lowering treatment prolonged for several months [115, 116].

In patients with familial hypercholesterolemia and familial combined hyperlipidemia, there is a CFR impairment, which in the latter case is also related to the phenotype [117, 118]. In asymptomatic patients with hypercholesterolemia, a reduced CFR has been as well observed, independently of the presence of CAD and directly related to the low-density lipoprotein levels [119–121]. As observed for diabetes, treatment is able to reverse the MBF abnormalities in these patients. Even short-term conditioning together with a low-fat diet has a favorable effect [122]. Specific lipid lowering therapy with statins improves adenosine MBF in otherwise healthy subjects with hypercholesterolemia only if they had reduced values before treatment [123]. A positive effect was as well registered in patients with familial hypercholesterolemia [124]. In CAD patients there is a delay in the CFR improvement as compared to the achievement of blood lipid normalization [125]. The CFR increase is detected mainly in the regions with abnormal reserve before treatment [126, 127]. However, in patients with diffuse atherosclerosis with angiographically normal coronary arteries, a disseminated MBF improvement has been registered [128]. Similarly, in a group of patients with angina, but without significant obstructions at coronary angiography, all affected by mild to moderate hypercholesterolemia, the baseline CFR significantly improves after 6 months of lipid lowering therapy; interestingly, there is also a reduction in anginal symptoms [129]. 

In otherwise healthy smokers, Campisi et al. have demonstrated preserved CFR under dipyridamole, but an abnormal response during CPT, which is more severe in case of more prolonged smoking habit [130]. Starting from the hypothesis that increased oxidative stress could be one of the mechanisms through which smoke causes vascular dysfunction, Kaufmann et al. have demonstrated a CFR improvement in smokers submitted to intravenous vitamin C administration [131]. The simple cessation of smoking restores a normal response to CPT in otherwise healthy subjects [132].

There are other factors that have been found to affect the MBF and CFR. For instance, Schindler et al. have observed a decreased response to CPT in patients with coronary risk factors and chronic inflammation detected by high C-reactive protein [133]. The same authors have registered an abnormal response to CPT in overweight and obese subjects and a decreased vasodilator CFR in the latter group only, suggesting that the endothelium dependent vasomotor function is more precociously affected than the total vasodilator capacity in patients with increased weight [134]. In postmenopausal women, long-term hormone replacement therapy has been found to improve the response to CPT only in those without associated coronary risk factors, whilst in those with a significant risk profile the reduced CFR under dipyridamole at baseline is not influenced by estrogens [135]. 

In summary, it appears clear that quantitative cardiac PET is a precious and reliable tool to investigate pathophysiologic changes in heart disease and to evaluate the possibility to prevent or correct them before they reach an otherwise detectable clinical expression. 

## 6. MBF and CFR in Patients with CAD

As regards the behavior of MBF and CFR in patients with CAD, there is a demonstrated relationship between the degree of coronary artery stenosis and the impairment of stress MBF and CFR, which is present independently of the employed perfusion tracer, as shown by studies performed with ^15^O-water, ^13^N-ammonia and more recently with ^82^Rb [136–140]. Another most interesting finding is that CFR in CAD patients is reduced as compared to healthy controls also in territories without epicardial stenosis [139]. Similarly, it has been demonstrated that the MBF and CFR in territories subtended by patent coronary artery by-pass grafts are lower than in normal controls, and this independently of the type of conduit (saphenous vein or internal mammary artery grafts) [141]. Conversely, it has been found that in case of coronary artery stenting there is a good early recovery of MBF and CFR, with values that are no significantly different from those of normal territories [142]. 

In territories with stenosis and perfusion defects in qualitative ^82^Rb PET, the MBF is abnormally reduced; however, low values are registered also in territories with angiographic stenosis but without apparent perfusion defects; moreover an inverse relationship between coronary stenosis and CFR has been registered [143]. It has been as well demonstrated that there is a relationship between stress MBF (and CFR) and the presence of symptoms and electrocardiographic changes [144]. There is a good agreement between the CFR values measured using quantitative PET and the flow velocity reserve measured using Doppler and transesophageal echocardiography [145]. PET can be useful to establish the effectiveness of coronary collateral circulation [146–148]. The expected reduction of MBF as compared to normal territories has been found to be much more severe in case of regional dysfunction, suggesting a protective role of well-developed collateral circulation [148].

## 7. The Additional Value of Quantitative Perfusion PET 

Previous studies comparing the visual assessment of myocardial perfusion using PET and SPECT had demonstrated the superiority of the former, chiefly because of the better quality of PET images and the sounder attenuation correction methodology [44, 53, 149, 150]. This superiority alone, however, would hardly justify the use of a more demanding, more expensive, and less widely available methodology, although some cost-effectiveness studies suggest that the higher accuracy of PET could compensate for the higher expenses [151, 152]. In particular, studying a wide patient population with matched pretest probability of CAD, Merhige et al. could demonstrate more than 50% reduction in invasive angiography and revascularization, 30% cost savings, and similarly excellent 1 year outcomes in patients evaluated with PET versus those examined with SPECT [153]. The scenario could become completely different if the added value of quantitative PET were taken into account. The advantage of MBF and CFR quantitation by PET over the simple evaluation of the relative tracer uptake, which may be performed either by PET or SPECT, must be examined separately in the various heart diseases.

## 8. Diagnosis of CAD

The current concept of diagnosis in patients with suspect CAD is to optimize the technique choice on the basis of the pretest probability. In general, therefore, a noninvasive imaging methodology finds its role in patients with intermediate pretest likelihood, because those with very low probability do not require imaging and those with high probability should directly proceed to invasive evaluation with coronary angiography [157]. The specific contribution of quantitative cardiac perfusion PET in this scenario is to help identifying the total ischemic burden in case of multivessel CAD and to pick patients with balanced three-vessel CAD, who are frequently missed using the relative tracer uptake, as it is performed in myocardial perfusion SPECT and even in qualitative perfusion PET [158–163]. By using previously identified stress MBF and CFR thresholds, Johnson and Gould have been able to construct a model that offers a high diagnostic reliability for an objective assessment of CAD burden of the single patient [144]. 

What is probably more important and certainly more innovative is the possibility to use quantitative perfusion PET to detect patients with preclinical CAD. As previously detailed, MBF and CFR have been found abnormal in subjects with various risk factor profiles. This circumstance and the fact that in many cases MBF and CFR monitoring may identify the degree of response to risk factor correction would support the use of quantitative PET for orienting primary prevention of CAD [164].

## 9. Evaluation of CAD Severity

 As already mentioned, the quantification of MBF and CFR increases the capability to detect multivessel coronary artery disease. More generally, there are sample data indicating that the hemodynamic meaning of a coronary stenosis is not easily predictable and that there is a wide variability of flow abnormalities for any given stenosis pattern [137, 138, 165]. Therefore, the quantification of MBF and CFR is much more reliable to predict the true myocardial perfusion than the relative tracer uptake in SPECT imaging. 

## 10. CAD Prognostication 

Independently of the definition of the CAD extent, a most important issue in current cardiology is to perform a correct and reliable prognostication of these patients. The prognostic value of myocardial perfusion PET has been already demonstrated using the simple visual assessment of myocardial uptake, thus with the same approach currently employed for SPECT perfusion tracers [166]. In particular, the superiority of a PET-based prognostication had been recognized even in patients with apparently normal perfusion pattern in ^201^Tl myocardial perfusion scintigraphy and in ^99m^Tc-sestamibi SPECT [167, 168]. The addition of gating during PET acquisition further improves the prognostic performance of ^82^Rb PET [169]. The abnormal MBF and CFR response to CPT has been shown to have prognostic implications [170]. With the more commonly used vasodilator stress, subsequent studies have shown that the quantitative assessment of MBF and of CFR has a clear prognostic meaning [171–174]. Most recently, a very large study has convincingly demonstrated that the global MFR has an incremental value for prognostication over all other established variables that can be derived from gated PET with ^82^Rb [175]. Patients in the lowest tertile of CFR have a significantly worse prognosis than the other subjects, independently of perfusion pattern and functional response to vasodilator stress. Conversely, patients with CFR in the highest tertile have a good prognosis even in case of perfusion defects and suboptimal functional response [175].

## 11. Detection of Myocardial Microvascular Disease

Another major contribution of quantitative myocardial PET has been to open the way to the assessment of myocardial microvascular disease. There is a wide range of cardiac conditions that imply, in a more or less central position for their physiopathology, the presence of a dysfunction in coronary microcirculation. According to a proposed classification, they can be grouped in four categories: type A includes coronary microvascular dysfunction occurring in the absence of obstructive epicardial coronary artery disease and other myocardial diseases; type B is the coronary microvascular dysfunction occurring in cardiomyopathies; type C covers coronary microvascular dysfunction occurring in the presence of obstructive CAD; finally, type D comprises iatrogenic coronary microvascular dysfunction [176]. The types A, C, and D, however, are someway related to CAD, because type A can be considered the equivalent of the early abnormalities in preclinical coronary artery disease, type C includes the reported abnormalities in territories not related to stenotic vessels, and type D the abnormalities in MBF that have been registered in patients submitted to percutaneous revascularization procedures. A very particular case of type D could be considered the cardiac allograft vasculopathy in heart-transplanted patients, because the process involves both the epicardial and the microvascular coronary system. In these subjects, recent data indicate that quantitative PET is able to identify (and potentially follow up) the cardiac allograft vasculopathy [177]. On the contrary, type B includes various different heart diseases in which the presence of microvascular dysfunction has been convincingly demonstrated mainly by quantitative perfusion PET. 

 The largest number of studies involves HCM patients. In these subjects, symptoms and signs that can mimic coronary artery disease are often registered, but mainly in the absence of any abnormality of the epicardial vessels [178, 179]. Conversely, the presence of microvascular impairment with reduced MBF under vasodilatation and decreased CFR has been demonstrated by several reports [180–182]. Moreover, various studies indicate that the microvascular involvement is a mainstay of HCM physiopathology and not just a late consequence of the increased wall thickness [183–186]. Accordingly and most importantly, the detection of microvascular dysfunction has major prognostic implications in these patients [187, 188]. The presence of microvascular impairment has been also demonstrated in patients with Anderson-Fabry disease, in whom cardiac involvement is a frequent complication [189, 190]. Similar findings have been observed in patients with idiopathic dilated cardiomyopathy (DCM) [191–195]. The prognostic implications of reduced stress MBF are present in DCM patients as well [196]. Other potential uses of quantitative PET in the patients with heart failure have been proposed [197, 198]. Although shown in a very preliminary study on a small patient population, the quantitative analysis of MBF appears promising to evaluate the effectiveness of medical treatment [199]. Finally, it has been demonstrated that in patients with hypertensive cardiomyopathy there is an impairment of microvascular function, which can explain the presence of anginal symptoms and is not homogeneously related with the degree of ventricular hypertrophy [200]. 

## 12. Hybrid Imaging

 The current standard of PET devices is to have an integrated CT scanner that allows attenuation correction and facilitates the anatomical interpretation of PET findings. This offers the advantage of permitting a simultaneous evaluation of myocardial perfusion and coronary vessel morphology by means of CT angiography, as has been demonstrated in several articles [196–207]. Other studies show that a CT aimed to estimate the calcium score can be used for attenuation correction in quantitative PET and therefore the two clinical data can be usefully combined [203, 208]. Only part of these studies, however, specifically evaluate the integration of PET quantitative perfusion parameters and CT results [204, 206, 207]. The main interest of this particular combination is to elucidate the complex relationship between coronary vessel abnormalities (beyond lumen obstruction and thus including wall atherosclerosis) and myocardial perfusion [209, 210]. Naturally these advantages must be weighed against the higher technical, logistical, and dosimetric burden of hybrid imaging [211]. 

## 13. Dosimetric Data

 The use of PET implies the administration of radioactive tracers. Therefore, a comment on the risks related to ionizing radiations is mandatory. The available data indicate that PET offers always a favorable dosimetric profile as compared to myocardial perfusion SPECT (Table 2) [154–156]. Among PET tracers, however, the use of ^82^Rb implies a much higher dose than ^15^O-water or ^13^N-ammonia. In case of hybrid imaging, the additional dose related to the CT procedure must be taken into account [211]. The most recent advances in this field, nevertheless, allow a significant reduction of the radiation dose for the CT and therefore justify the growing trend to consider hybrid imaging as a most desirable approach for perfusion PET [212, 213]. 

## 14. Comparison with Other Imaging Modalities

 There are no doubts that PET is the most established technique for MBF and CFR measurement. Given the importance of these quantitative parameters, there is a growing interest for the possibility to obtain them using other imaging modalities that are already highly effective for the assessment of heart disease. In particular, the capability of magnetic resonance imaging to achieve reliable MBF using a first-pass technique during gadolinium injection has been demonstrated [214]. Although it requires quite complex computational approaches, this is probably the most effective alternative to PET, with the major advantage of not requiring ionizing radiation, but with other limitations related to the general contraindications for MRI in some patient groups, such as subjects with pacemakers or with renal disease [215]. Conversely, the feasibility of MBF quantitation using CT angiography has been more recently demonstrated, but dosimetric problems appear a major hindrance to the widespread use of this modality [216, 217].

## 15. Future Developments and Challenges

 The most interesting coming development for cardiac PET is the possibility to use an ^18^F labeled tracer for perfusion imaging. The compound ^18^F-flurpiridaz is already under advanced evaluation and phase 3 studies are ongoing [35, 218, 219]. The image quality is excellent and the results of the preliminary reports of phase 1 and phase 2 suggest that this tracer could be a major advance in perfusion PET [35, 218, 219]. The prolonged half-life of ^18^F opens the way for centers without on-site cyclotron to purchase flurpiridaz from a vendor, and therefore increases the number of potential users. It makes as well possible to inject the tracer during dynamic exercise using the standard equipment (either bicycle ergometer or treadmill), with the advantage of a physiologic stimulation instead of a pharmacologic one. On the other hand, this last procedure would preclude the quantitation of MBF, because of the impossibility to acquire a dynamic PET during tracer injection. Preliminary data suggest that a quantitative assessment of MBF using flurpiridaz is feasible and reliable [220–222]. Therefore, the availability of this tracer will open new perspectives, but the difficult choice between performing exercise PET and obtaining MBF quantitation has to be made. 

 The tracer problems that flurpiridaz could partly overcome are not the sole hurdles to the definitive acceptance of quantitative PET in the clinical routine. Most of these obstacles, however, are in general related to the disadvantages of PET versus SPECT for the clinical practice and are also influenced by the heterogeneous logistic, regulatory, and economic situations in the different countries [11, 25]. For instance, in USA the cardiologists are frequently directly involved in the execution of imaging techniques using radioactive tracers; this has driven a major growth in the number of nuclear cardiology exams and could increase the clinical demand of more effective approaches [223]. On the other hand, the large number of office-based practices could be an obstacle to the widespread use of PET, because this technique is certainly more demanding than SPECT and requires large investments [11]. Conversely, in Europe the execution of nuclear cardiology is usually limited to the nuclear medicine specialist, and most exams are performed in hospitals [224]. This could adversely influence the progress of cardiac PET because the interest for cardiology in nuclear medicine departments is often scarce, and the pressure of the clinical demand more limited [223]. However, the circumstance that PET scanners are already available in most nuclear medicine laboratories would facilitate the choice of shifting cardiology from SPECT to PET [25]. 

The specific problems concerning the quantitative approach are the more complex acquisition procedure, the greater computational burden, and the need of dedicated processing software. List mode acquisition capabilities for dynamic studies, however, are not a major problem in most modern PET scanners. The implementation of reliable programs for image processing and subsequent MBF quantitation directly in the PET scanner software by the producers would be highly desirable, and would drastically reduce the difficulties encountered so far, making the whole procedure more easily acceptable for the physician. Unfortunately, the demand is still too limited for obliging the vendors to make this choice. Ongoing studies, which compare the available programs and models in order to identify the most reliable, could be helpful for reducing the current uncertainty about the ideal approach. 

## 16. Final Remarks

 The usefulness of quantitative PET in heart disease has been convincingly proven. However, every new technique must find its place in the clinical scenario through the demonstration of its cost/effectiveness and of its superiority over already established methodologies. In case of quantitative PET, large prospective studies focused on defining its relative merits in a direct comparison with other approaches are still lacking. The problem involves more in general the role of noninvasive diagnostic techniques and some important trials are trying to clarify this issue, but reaching definitive evidence will still take time, and unfortunately the chosen approach for PET evaluation does not include quantitative measurements [225, 226]. On the other hand, there are no doubts that the technical and clinical prerequisites for a widespread use of quantitative PET have already been fulfilled. What is probably most important nowadays is that the cardiologic community becomes aware of the potential advantages of quantitative PET and start considering its use. The nuclear medicine physician, conversely, must improve the availability and reliability of quantitative cardiac PET, so that demand and offer may match and increase together. If always performed having in mind the patient benefit, this dialogue will certainly lead in a reasonable time to the realistic definition of the true role of quantitative cardiac PET.

## Figures and Tables

**Table 1:** Comparison of the features of the main PET myocardial perfusion tracers.

Tracer	Patient throughput	Availability	Image quality	Background	Quantitation
H_2_^15^O	High	Low	Very poor	NA	Optimal
^ 13^NH_3_	Medium	Medium	Fair to good	Quite high	Good
^ 82^Rb	High	High	Fair to good	Acceptable	Fair
^ 18^F-agents	Low	High	High	Low	Good

**Table 2:** Comparison of radiation doses for various cardiac diagnostic procedures.

Technique	Dose, mSv
Thallium-201 SPECT	25.1–31.4
Technetium-99m-sestamibi SPECT	10.6–12
^ 82^Rb PET	13.5–16
^ 13^N-ammonia PET	2.4
^ 15^O-water PET	2.5
CT angiography (64 MSCT)	8.4–21.4
Coronary angiography	2.3–22.7

Legend: MSCT: multislice computed tomography; mSv: millisievert; PET: positron emission tomography; SPECT: single-photon emission computed tomography. Ref: [154–156].
